# Valorisation of Chitosan Natural Building Block as a Primary Strategy for the Development of Sustainable Fully Bio-Based Epoxy Resins **

**DOI:** 10.3390/polym15244627

**Published:** 2023-12-06

**Authors:** Iolanda Fusteș-Dămoc, Roxana Dinu, Teodor Măluțan, Alice Mija

**Affiliations:** 1 University Côte d’Azur, Institute of Chemistry of Nice (ICN), UMR CNRS 7272, 06108 Nice, France; 2 “Cristofor Simionescu” Faculty of Chemical Engineering and Environmental Protection, “Gheorghe Asachi” Technical University of Iasi, 73 Prof. D. Mangeron Street, 700050 Iasi, Romania

**Keywords:** chitosan, biopolymer, thermosets, materials, epoxidized vegetable oil

## Abstract

The non-toxic and biodegradable nature of chitosan makes it a valuable resource offering promising opportunities in the development of bio-based materials with enhanced mechanical and thermal properties. In this work, the combination of epoxidized linseed oil, oxalic or citric acids, and chitosan (CHI) as a curing accelerator presents an attractive strategy to create bio-based and sustainable thermosetting materials. This article aims to provide a comprehensive exploration of the systems reactivities, characteristics, and performance evaluation of the designed bio-thermosets. Both the nature of the two carboxylic acids and the presence of chitosan are shown to have a big impact on the thermomechanical properties of the developed networks. While oxalic acid favours the formation of elastic networks, with low *T*_g_ values (increasing with CHI content between 0.7 and 8.5 °C) and relatively low Young’s modulus (~2.5 MPa), citric acid promotes the formation of very dense networks with lower mass of the segments between the crosslinks, having 20 times higher *T*_g_ values (from 36 to 45 °C) and ~161 times higher Young’s modulus (from 94 MPa up to 404 MPa in these systems). The CHI has a strong impact on the curing reaction and on the overall properties, by increasing the materials’ performance.

## 1. Introduction

The increasing global concern for environmental sustainability has driven significant research efforts towards developing eco-friendly materials that can replace their conventional petrochemical-based counterparts. Thermosetting materials play a crucial role in modern industries due to their exceptional mechanical properties, chemical resistance, and thermal stability. However, the conventional thermosets, predominantly non-recyclable and non-biodegradable, pose environmental and sustainability challenges. In this context, the synthesis of bio-based and sustainable thermosets using renewable resources has emerged as a key area of research for achieving a more sustainable and circular economy.

In recent years, significant attention has been devoted to exploring the potential of epoxidized vegetable oils as renewable precursors for thermosetting resin production. Epoxidized vegetable oils (EVO) have gained attention due to their non-volatile, eco-friendly and sustainable nature [1]. The industrial interest in vegetable oil-based epoxy resins has increased significantly as they offer a safer alternative to toxic or carcinogenic bisphenol A precursors [2,3]. These EVOs can undergo thermal, anionic, or cationic (co)polymerization with various compounds to produce thermosetting bio-based materials, ranging from elastomers to rigid and tough plastics. Vegetable oils such as linseed oil, castor oil, and tung oil, containing hydroxyl or epoxy groups in their fatty acid molecules, have been extensively studied for this purpose [4,5]. EVOs have also the advantage to be obtained through a green epoxidation process [6]. The epoxy groups in the EVOs structure can be cured using suitable curing agents, under appropriate conditions. The choice of the curing agent depends on the desired application of the final thermoset [7]. Hardening agents such as anhydrides or carboxylic acids have been used for crosslinking EVOs, resulting in materials with good thermo-mechanical properties [1].

Epoxidized linseed oil (ELO), derived from flaxseed, has provoked particular interest due to its abundance, low cost, inherent biodegradability, and excellent thermal stability. The presence of ~5.5 epoxy groups per triglyceride enables crosslinking reactions, leading to the formation of highly crosslinked thermosets. Various hardeners, including C6-C16 diacids [8], cyclic anhydrides [9], and tannic acid [10], have been utilized together with many others for crosslinking the epoxidized linseed oil.

In addition to vegetable oils, other renewable raw materials have gained interest in the development of bio-based materials. Among these materials, chitin, the second most abundant polysaccharide in nature after cellulose, has attracted attention due to its availability and functionality [11]. Chitin is primarily extracted from the exoskeleton of crustaceans and molluscs, and large amounts of chitin are generated as by-products in the seafood industry, with only a small part being valorised, and the rest considered waste [12]. Alternative sources of chitin, such as fungi, insects, fungal bodies, and microbial biomass, also exist, but their production costs are typically higher compared to crustacean-based chitin. Shrimp shell-based chitin currently occupies about 80% of the total chitin market [13].

As a polysaccharide derived from chitin, chitosan exhibits a chemical structure consisting of *β*-(1→4)-linked D-glucosamine and N-acetyl-D-glucosamine units (Figure S1). The presence of amino functions in the deacetylated units at the C-2 position, and of hydroxyl groups at the C-3 and C-6 positions, grants chitosan remarkable reactivity and versatility in material chemistry. These functional groups serve as active sites for chemical modifications, crosslinking reactions, and interactions with other components, making chitosan a valuable building block for the development of sustainable and high-performance thermosets [14].

By modifying the structure of chitosan with hydrophilic or lipophilic molecules, its solubility in organic solvents or acidic solutions can be improved [15]. Chitosan is typically soluble in acidic solutions with a pH < 5.5, where the amino groups are protonated and carry a positive charge [16,17,18,19,20,21]. Its non-toxicity, biodegradability, and biocompatibility make chitosan suitable for various applications, including biomedical, pharmaceutical, and food industries [14].

Chitosan has been used in some, but still few, thermoset systems. Nepomuceno et al. [22] developed thermoset resins foreseeing potential applications such as curative bio-composites using epoxidized soybean oil cured with salicylic acid and chitosan in different mass percentages: 1, 3, and 5 wt.%. Furthermore, through RAFT polymerization of a chitosan derivative and epoxidized tung oil, and using citric acid as a curing agent, Song et al. developed materials for packaging applications [23].

Jabeen et al. [24] synthesized blended systems based on chitosan in 2 wt.% acidic aqueous solution and diglycidyl ether of Bisphenol F resins (BFDGE) solution in acetone. The blend films were obtained through solvent casting. Wu et al. [25] reported synthesizing elastomers through Diels–Alder reactions between furfural-modified chitosan and furfural-modified epoxy natural rubber. These materials exhibited excellent mechanical, self-healing, recyclable, and antibacterial properties, good performance for 4D printing, smart materials for medical equipment, and others. Less elastic materials were prepared by Kanthiya et al. [26], who blended chitosan with diglycidyl ether of bisphenol A and epoxidized natural rubber. The obtained materials showed a decrease in glass transition from 60 °C for the one without chitosan to 51 °C for the one with 20 wt.% chitosan, but also a decrease in tensile strength and elongation at break when the percentage of chitosan increased. However, the systems revealed improved properties for use in packaging, medical, and agricultural applications. Also, for biomedical applications, Chen et al. [27] developed a new biodegradable stent made with crosslinked films of chitosan and ethylene glycol diglycidyl ether. This stent had a shape-memory property when immersed in PBS solutions at different pHs, and in vivo studies showed no thrombus formation in the stent-implanted vessel. Thereby, this stent represents an alternative to metal stents and can also be used as a vehicle for local drug delivery.

In this study, the aim is to develop new thermosetting materials by combining only natural synthons and derivatives: epoxidized linseed oil and low-molecular-weight chitosan. The potential of these novel thermosets to replace conventional petrochemical-based counterparts, mitigating environmental impact and fostering a circular economy by enabling the recycling of chitin resources, is considered. To enhance system reactivity and network crosslink density, natural and non-toxic acids, namely oxalic and citric acid, were added. Oxalic acid, a naturally occurring compound found in various plants, exhibits high reactivity due to its low acidity constants. Citric acid, produced from Aspergillus Niger mold cultures, has been efficiently used in reactions with epoxidized vegetable oils [28,29]. The chemical structures of the reactants are depicted in Figure S2.

Oxalic acid has very low acidity constants and its solubility in water is almost two times lower than in ethanol. Thus, its form in ethanol is more stable and its oxalate ions are less stable. In contrast, the solubility of citric acid in water is equal to that in ethanol. These two acids present a very good reactivity due to their nature: oxalic acid is a dicarboxylic acid with (pKa): 1.27; 4.27; and citric acid is a tricarboxylic acid with pKa_1_ = 3.13, pKa_2_ = 4.76, pKa_3_ = 6.39 [29]. To design the networks, we hypothesized that the primary amines and/or the –OH functions in the chitosan structure can cleave the oxirane rings through nucleophilic addition reactions and act as hardeners. Moreover, the presence of carboxylic acids increases the reactivity and selectivity of chitosan [11].

By comparing the effects of a dicarboxylic acid (oxalic acid) and a tricarboxylic acid (citric acid), we aim to evaluate their respective contributions to the properties of bioresins.

The study investigates the influence of varying composition and processing parameters on the resulting material properties, including mechanical strength, thermal stability, chemical resistance, and other relevant characteristics. The findings of this research will contribute to the advancement of sustainable thermosetting systems and their potential applications.

## 2. Materials and Methods

### 2.1. Materials

Epoxidized linseed oil (ELO; average molar mass = 980 Da; average functionality = 5.5 epoxides per triglyceride, viscosity~870 cPs/25 °C) was supplied by Valtris Specialty Chemicals (Manchester, UK). Chitosan (CHI; low molecular weight = 50–190 kDa; 84% deacetylated), oxalic acid anhydrous (OA; ≥99.0%), citric acid monohydrate (CA; ≥99.0%), and ethanol (EtOH; absolute, ≥99.8%) were purchased from Sigma Aldrich (Saint-Quentin-Fallavier, France). All chemical compounds were used as received without any further purification.

### 2.2. Samples Preparation

Formulations of the ELO/CHI//OA and ELO/CHI//CA mixtures were prepared in the presence of ethanol as the solvent (~40%). ELO/CHI//OA and ELO/CHI//CA mixtures were prepared using epoxy: acid molar ratio R = 1, and CHI was added in different mass percentages: 4%, 3% and 2%. Hence, the acronyms used for these 8 systems are given in the Table 1, labelled based on their ratios and mass percentages.

To prepare the bio-resins, the selected amounts of OA or CA were firstly dissolved in ethanol at around 50 °C and then the established amounts of CHI and ELO were added. The mixtures were stirred for about one hour, at room temperature, and the ethanol was removed under vacuum. The mixtures were poured into molds and placed in a convection oven for 1 h at 80 °C and 2 h at 150 °C for curing, and 0.5 h at 180 °C for post-curing. The freshly prepared formulations were analysed using in situ FT-IR and DSC to follow the copolymerization reaction. The curing and post curing program were previously determined through DSC analysis.

### 2.3. Fourier Transform Infrared Spectroscopy (FT-IR)

The kinetics of the crosslinking reactions were performed in situ with a Thermo Scientific Nicolet iS50 FT-IR spectrometer in attenuated total reflectance (ATR) mode. The spectra of raw compounds and of completely cured resins were registered at room temperature. Also, the structural evolution of the systems during crosslinking was investigated by scanning the fresh mixtures under dynamic heat from 30 °C to 180 °C at a heating rate of 10 °C min^−1^. The absorption bands were collected in the range 600–4000 cm^−1^ with a resolution of 4 cm^−1^ and 64 scans. The data were analysed using the OMNIC 9.8 software. The conversion of the functional epoxide and carboxyl groups at time (*t*) is denoted as (%) and defined by Equation (1):

(1) $$\%=\frac{{\left(\frac{A_{functional\;groups}}{A_{ref}}\right)}_{0}-{\left(\frac{A_{functional\;groups}}{A_{ref}}\right)}_{t}}{{\left(\frac{A_{functional\;groups}}{A_{ref}}\right)}_{0}}\times 100$$

where the area absorbance peaks calculated at the initial time (*A*_0_) and at different times (*A_t_*), *A_ref_* is the reference band that was considered in this study at 1460 cm^−1^ corresponding to the scissoring of CH_2_, asymmetric bending of CH_3_ of the ELO structure; *A_functional groups_* was taken for the epoxy group at 826 cm^−1^. For the carboxylic acids group (hydrogen bonded O-H out of plane-bending vibration), the reference was taken for the reactive mixtures at 878 cm^−1^, even if this band appears in the individual acids at 897 (CA) and 792 cm^−1^ (or it is covered by a large band centred for OA at 1166 cm^−1^) Probably, in the reactive mixture, the H-bondings created between the O-H with O or N heteroatoms from ELO or CHI structures provoked a shifting in frequencies of absorption of this band [30].

### 2.4. Differential Scanning Calorimetry (DSC)

The DSC measurements were performed on a Mettler Toledo (Viroflay, France) DSC 3 apparatus, controlled using STARe 16.10 Software. The temperature and heat flow of the instrument were calibrated at 3 points, using water, indium, and zinc standards. The reactivity of freshly prepared mixtures of ELO/CHI//OA and ELO/CHI//CA were performed directly in 40 μL aluminium crucibles, under a heating rate *β* of 10 °C·min^−1^ and in a temperature range of 25–210 °C.

### 2.5. Thermogravimetric Analysis (TGA)

TGA measurements were realized with a Mettler Toledo (Viroflay, France) TGA 2 equipment. Samples of about 10 mg were placed into 70 μL alumina pans. To characterize their thermal stability, cured samples of the thermosets were heated at 10 °C·min^−1^ from 25 °C to 1000 °C under oxidative (air) gas flow of 50 mL·min^−1^.

### 2.6. Dynamic Mechanical Analyses (DMA)

DMA experiments were conducted on a Mettler-Toledo DMA 1 instrument, equipped with STAR 16.10 software, using a dual cantilever fixture. Storage modulus values (*E*′) and damping factors (tan *δ*) were measured at a heating rate of 3 °C·min^−1^ from −150 °C to 150 °C at 1.0 Hz frequency. The *α* relaxation (glass transition) was assigned as the maximum damping factor (tan *δ* = *E*″/*E*′).

The crosslink density of the samples with OA and CA was calculated from the value of the elastic modulus in the rubbery state by Equation (2), according to Flory’s theory [31]:

(2) $$\nu =\frac{E^{\prime }}{3RT}$$

where *E*′ is the storage modulus of the thermoset in the rubbery plateau region at *T*_g_ + 70 °C, *R* is the universal gas constant, and *T* is the absolute temperature in Kelvin.

### 2.7. Shore Hardness Tests

The hardness of the thermosets was determined using a ZwickRoell (Ars-Laquenexy, France) 3116 device. A Shore A device was used for both ELO/CHI//OA and ELO/CHI//CA thermosets. The loading force that pressed on the samples was 12.5 N ± 0.5 N and 50 N ± 0.5 N, respectively. The presser foot had a firm contact with the tested materials. Each measurement was repeated six times for each sample and the values averaged.

### 2.8. Tensile Tests

Using an Instron 3365 universal test machine (Norwood, MA, USA) and BlueHill Lite 4.21 software, the tensile properties of the bio-resins were determined. Five dog bone type V samples were evaluated for each system at a speed of 5 mm·min^−1^. The data thus obtained were averaged.

### 2.9. Density

The density of the developed systems was determined based on the mass/volume ratio. Three specimens with the dimensions of 50 × 5 × 2 mm^3^ for each formulation were analysed, the final result being considered their average.

## 3. Results and Discussions

**Scheme 1:**
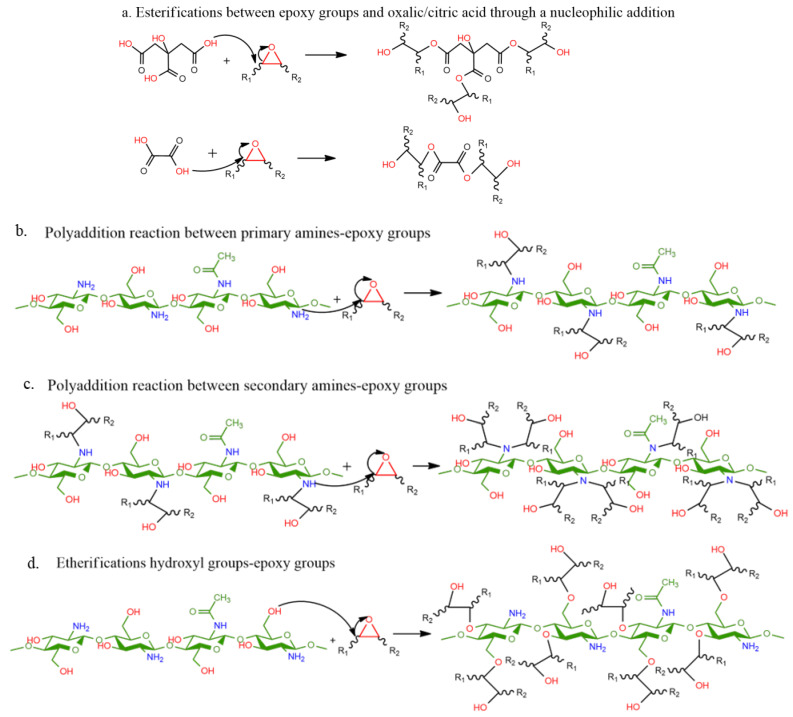
Potential reactions that can occur in ELO/CHI/acid mixtures.

The chemical reactions between the functional groups present in the developed ELO/CHI/acids formulations, such as epoxy, carboxyl, hydroxyl, and amine groups, are quite complex, implying several potential reactions of addition, esterification, and etherification (Scheme 1). A first hypothesis is the fact that epoxy groups react with oxalic/citric acid through a nucleophilic addition to form esters (Scheme 1a). Also, the amino groups in the structure of chitosan can interact with the carboxylic groups to form intermolecular or intramolecular chains. Moreover, the polyaddition reaction of chitosan’s primary amines with the epoxy groups present in ELO could lead to the formation of secondary amines that further react with the epoxy groups present in the media (Scheme 1b,c) [29]. During this addition, hydroxyl groups are formed and, together with the –OH groups already existing in the chitosan structure, can in some conditions (high temperature, excess of epoxy functions, etc) result in etherification with the epoxies (Scheme 1d).

The hydroxyl groups also have an autocatalysing effect, increasing the rate of main amine–epoxy additions [7].

### 3.1. Fourier Transform Infrared Spectroscopy (FT-IR)

From Figure S3a, the principal FT-IR absorption peaks for the chitosan are at 3361 cm^−1^ and 3287 cm^−1^, attributed to the N–H and O–H stretching. The peak at 1649 cm^−1^ corresponds to the amide band I C=O, while at 1554 cm^−1^ it corresponds to the amide band II –NH_2_ due to deacetylation. The absorption peak characteristic to the symmetric and asymmetric stretching vibration of the C–O–C bridge from the saccharide structure of chitosan appears at 1153 cm^−1^ (Table S1) [2,32].

From the FT-IR spectrum of ELO (Figure S3b and Table S1), we can observe the characteristic absorption peaks at 3497 cm^−1^ for –O–H stretching (weak); at 2920 cm^−1^ for –C–H, asymmetric stretching of CH_2_, CH_3_; at 2851 cm^−1^ for –C–H, symmetric stretching of CH_2_, CH_3_; and at 1743 cm^−1^ for triglyceride carbonyl of ester C=O. The asymmetric C–O stretching of ester groups (O=C–O) appears at 1152 cm^−1^ and the C–O stretching of O–CH_2_ appears at 1098 cm^−1^. At about 847–826 cm^−1^, the absorption peaks are attributed to the C–O deformation of the oxirane group [1,8].

The common peak assignments of the two selected acids OA and CA are represented by the wide bulb absorption band characteristic of the O–H bond (COOH) at 3430–2500 cm^−1^ (for OA) and at 3500–2500 cm^−1^ (for CA), and by the C=O stretching mode in the carboxylic dimer at 1750 cm^−1^ and 1689 cm^−1^ (for CA) and at 1689 cm^−1^ (for OA). In the case of CA, the presence of a peak at 1207 cm^−1^ can be observed, which characterizes the C-O group (Figure S3d, Table S1). The out-of-plane O–H bending bands of carboxylic acids occurs at 897 (for CA) and 792 (for OA) cm^−1^, respectively (Figure S3d, Table S1).

**Figure 1:**
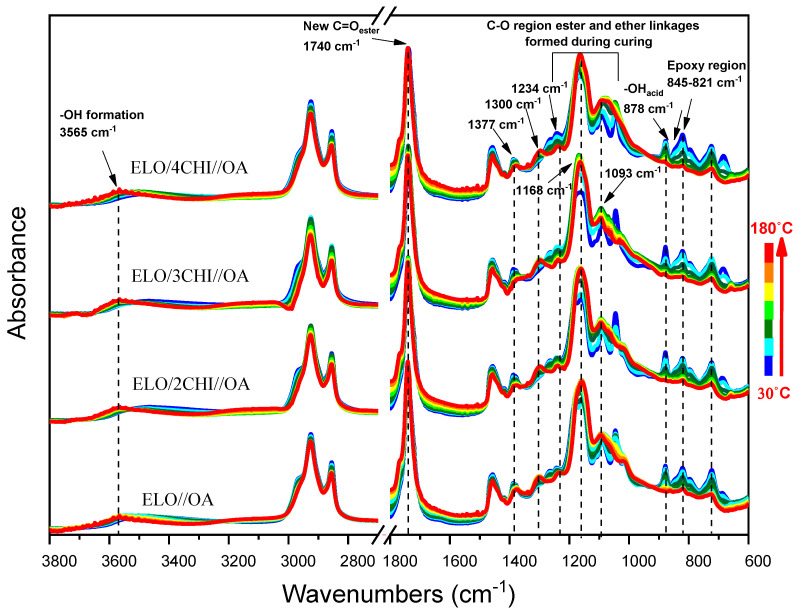
Evolution of FT-IR spectra during heating from 30 to 180 °C. Comparison of formulations with OA: ELO//OA; ELO/2CHI//OA; ELO/3CHI//OA; and ELO/4CHI//OA.

Figure 1 presents the FT-IR spectra of the four fresh mixtures of ELO/CHI and OA heated from 30 °C to 180 °C. Evaluating the systems’ reactions and comparing their reactivity using in situ FT-IR analysis, the shift of the peak from 3361 cm^−1^ (from chitosan) to 3565 cm^−1^ for the O-H formed during cross-linking is observed. The absorption band of this peak decreases in intensity, showing that the amino groups react with the epoxy and carboxyl groups. Each system has two peaks in the 3000–2800 cm^−1^ region for the asymmetric stretching of CH_2_, CH_3_ and the symmetric stretching of CH_2_, CH_3_. The peak at 1740 cm^−1^, corresponding to C=O ester group, increases in intensity for all four mixtures during heating, a sign that the ELO ratio favours the esterification reactions. It is seen that the 1377 cm^−1^ peak from the amide band III of chitosan gradually shifts towards the final temperature, which proves that it reacted even with N from the amide. Moreover, the absorbance bands for the primary and secondary hydroxyl groups from chitosan at 1023 cm^−1^ and at 1057 cm^−1^ join in a single broad peak at 1093 cm^−1^, and the peak at 1153 cm^−1^ for ethers moves to 1168 cm^−1^, indicating that new ester groups were formed during heating. The peaks of 821 cm^−1^ and 845 cm^−1^ for the oxirane rings and 878 cm^−1^ for the hydroxyl groups of the carboxylic groups disappear completely, which means that these groups reacted as we assumed in Scheme 1. At 1300 cm^−1^, we can observe β (OH) symmetric stretching from oxalic acid which increase with temperature. All fresh mixtures containing OA show the same changes, as illustrated in Figure 1.

**Figure 2:**
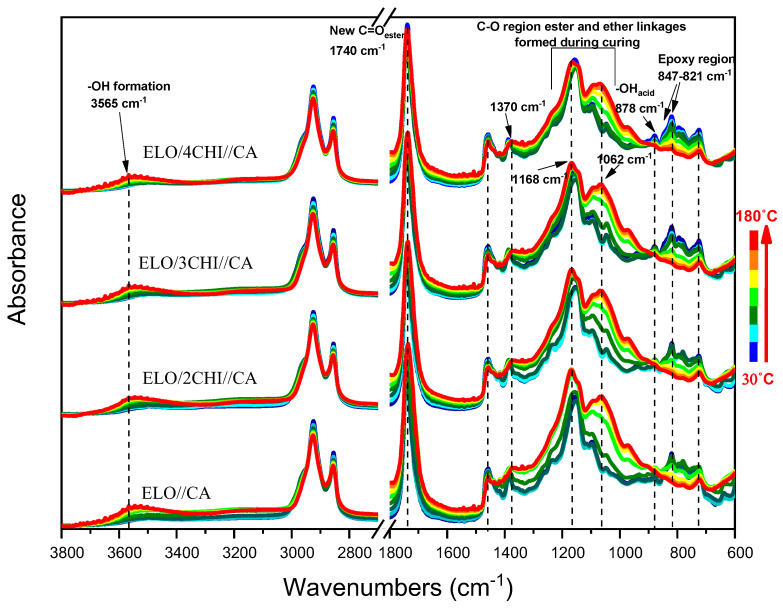
Evolution of FT-IR spectra during heating from 30 to 180 °C. Comparison of formulations with CA: ELO//CA; ELO/2CHI//CA; ELO/3CHI//CA and ELO/4CHI//CA.

**Figure 3:**
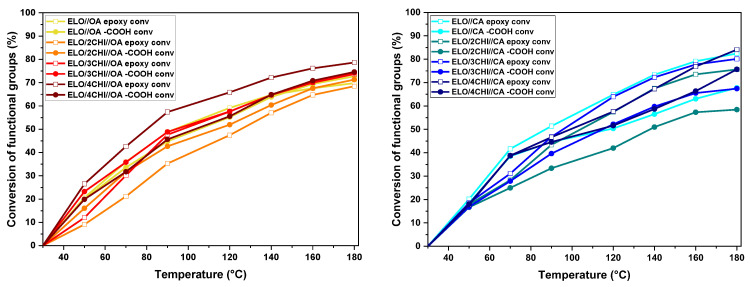
Conversion of functional groups (%) as a function of temperature for the ELO/CHI//OA and ELO/CHI//CA systems.

Figure 2 displays the evolution of the FT-IR spectra of ELO/CHI mixtures with CA during dynamic heating from 30 °C to 180 °C. As in the case of OA systems, we can observe a shift of the 3361 cm^−1^ peak in the chitosan structure up to 3560 cm^−1^, attributed to O–H stretching (Table 2). Also, compared to OA systems, the peak at 1062 cm^−1^ is much higher, showing a shoulder at 1093 cm^−1^, which highlights the appearance of more esters and ethers as a result of esterification reactions between the carboxylic groups and epoxy groups, the citric acid having three carboxylic groups. In the case of these mixtures, the epoxy and carboxylic groups completely disappear at 180 °C, indicating that at this temperature the groups’ conversion is accomplished. In most of the samples, the decrease at approximately the same rate of –COOH absorption at 878 cm^−1^ and epoxide absorption at 821 cm^−1^ occurs at the same time (Figure 3).

The FT-IR spectra collected at 180 °C for the developed systems (Figure S4) are more or less alike, one of the differences being related to the appearance of the peak at 1238 cm^−1^ attributed to –O–C(O)–C stretching of citric acid triesters, generated during heating, where the three carboxyl groups reacted. It can also be observed that the appearance of the peak at 1300 cm^−1^ in the case of OA systems, attributed to the symmetric β (OH) stretching, was also generated during heating.

The evaluation of the conversion of functional groups relative to temperature is shown in Figure 3. We can see that the peak intensity at 821 cm^−1^ for the epoxy groups decreases concomitantly with the decrease of the peak for the carboxyl group (–OH in acid) at 878 cm^−1^. It was found that for the ELO/CHI//OA systems, the reactions occurred in much closer proportions, about 62–79%, (Figure 3), considering the high reactivity of OA.

In contrast, the degrees of conversion as a function of temperature were more different for the ELO/CHI//CA systems, between 60 and 85%, with lower conversions of acid to epoxy groups being noted. This may be due to the competing reactivities of the amino and hydroxyl groups in the chitosan structure, respectively, which entered into the distinct types of reactions proposed above in Scheme 1.

It can be noticed that the conversions of citric acid systems heated up to 180 °C reach higher values than those with oxalic acid, a key factor being that citric acid has three carboxyl groups, while oxalic acid has only two carboxyl groups.

### 3.2. Differential Scanning Calorimetry (DSC) Studies

**Figure 4:**
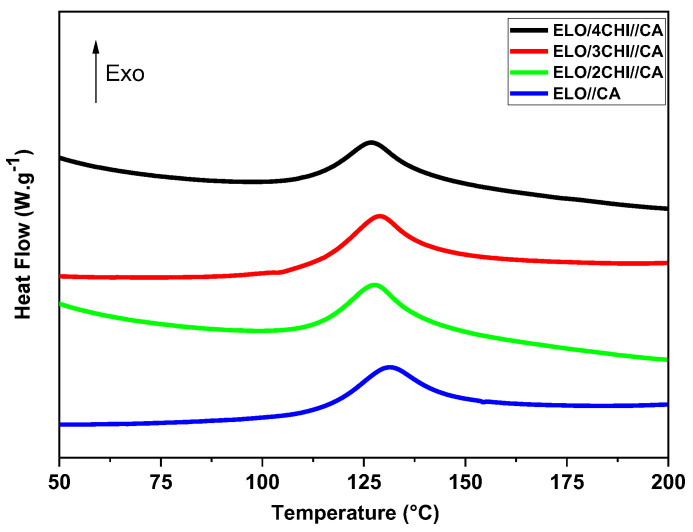
Dynamic DSC thermograms for ELO//CA systems with different percentages of chitosan.

Figure 4 and Figure S5 shows the DSC thermograms of the epoxy mixtures. Both the thermal enthalpy curves of the neat ELO//acid-based resins and those with different mass percentages of chitosan are characterized by a distinctive exothermic phenomenon with a single temperature peak.

The curing process starts, in most cases, immediately after the dicarboxylic acids melt. The melting transition of citric and oxalic acids was not as obvious, probably due to its higher initial reactivity during the premixing step [8]. It can be seen that, in the case of ELO/CHI//OA systems, the reactions start at 32–56 °C and are very fast, unlike ELO/CHI//CA systems, which start at 94–100 °C and end at almost 178–192 °C. The obtained results show that the reaction’s peak is shifted gradually to lower temperatures with the addition of chitosan. The OA systems presented lower maximum temperatures (*T*_peak_ = 82–87 °C) than CA systems, where the *T*_peak_ were occurring in the interval 127–131 °C.

It can also be seen that the height and area of the exothermic event increase with chitosan loading, indicating the improvement of the thermal behaviour of the systems. The values obtained from the calculation of the conversion degrees correlate very well with the results obtained for the enthalpies from the DSC studies (Table 3). Also, the difference in enthalpies of citric systems (125 J·g^−1^), which are higher than oxalic systems (50 J·g^−1^), can be found in their conversions. The enthalpy values obtained for both oxalic and citric acid samples increase significantly with the addition of CHI mass percentage.

The highest value for the enthalpy of reaction was obtained for the ELO/4CHI//CA system with 173 J·g^−1^, compared to ELO/4CHI//OA, where 107 J·g^−1^ was obtained. This observation can be attributed to the higher reactivity of citric acid, which is a tricarboxylic acid, compared to oxalic acid, which is dicarboxylic. Surprisingly, the lowest enthalpy values were obtained for the systems without chitosan, being about 125 J·g^−1^ for the ELO//CA system and 50 J·g^−1^ for the ELO//OA formulation. In conclusion, the addition of chitosan improves the chemical reactivity of the systems by actively participating and chemically binding in the polymeric network of the designed thermosetting materials.

### 3.3. TGA Studies

**Figure 5:**
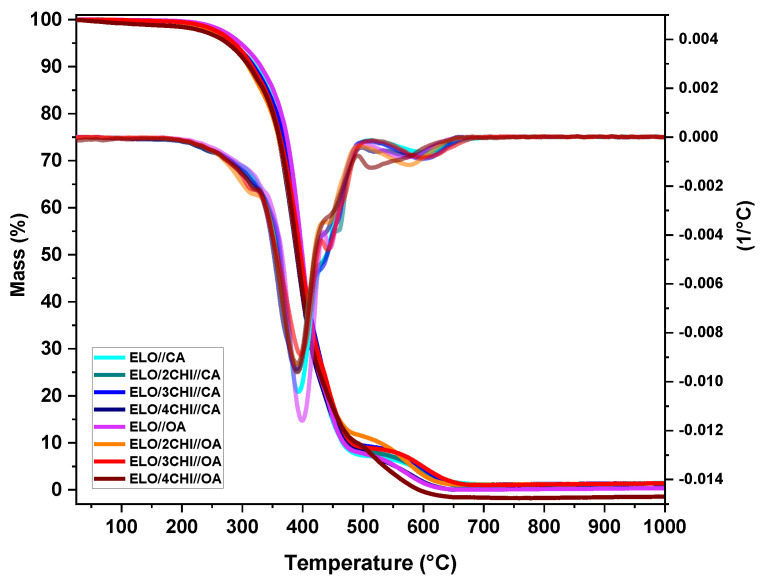
TGA and DTG curves of the ELO-based systems crosslinked with OA or CA and loaded with different mass percentages of chitosan.

Thermal stability and the decomposition behaviour of the obtained samples were investigated under air atmosphere through thermogravimetric analysis. The TGA curves, as well as the related derivatives depending on the temperature, are plotted in Figure 5.

Thermal behaviour of both raw resins and systems loaded with different percentages of chitosan showed a two-stage process. For neat chitosan (Figure S6), the thermal degradation is divided into three distinct steps. The first decomposition stage ranges between 35–150 °C, with a maximum degradation at about 55 °C and a mass loss of ~7%. This first stage can be associated with the evaporation of moisture present in chitosan. The second and the major step (mass loss~52%) is ranged between 200–400 °C and is characterized by the elimination of side groups and the cleavage of the glycosidic bond of chitosan. In the last stage (420–750 °C), the mass loss of approximately 38% can be attributed to the destruction of the pyranose rings and the oxidative degradation of the compound [2,24].

The thermal degradation behaviour of the developed thermoset systems with or without chitosan is divided in two stages. The first step is characterized by a complex peak between 200–500 °C with a maximum mass loss around 380–400 °C. In this major step, the mass loss is about 89–93% and it can be attributed to thermal decomposition. There is a hemolytic rupture of the covalent bond in the polymeric structures and a formation of radicals that recombine with oxygen in the air. In the second stage, the thermo-oxidative degradation takes place, at which point highly reactive peroxides are formed, after which the thermal decomposition process is completed, and the residual char left behind. The second stage is ranged between 500 °C and 700 °C, where about 6% of the mass is lost.

The degradation temperature was established as the temperature at which the materials lose 5% of their mass (*T*_5%_), the obtained values being plotted in Table 4. Regarding the thermal stability of the neat resins, it can be observed that the value obtained for ELO//OA (*T*_5%_ = 297 °C) is higher compared to that of ELO//CA (*T*_5%_ = 283 °C). Considering the influence of chitosan on the thermal stability of the systems, a slightly different behaviour can be observed depending on the nature of the acid used in the materials’ composition. For the systems crosslinked with CA, the addition of chitosan does not affect their thermal stability at all, except for the system with the largest amount of chitosan (ELO/4CHI//OA: *T*_5%_ = 276 °C), where the thermal stability is slightly reduced by 7 °C. In the case of the OA-based system, from the beginning, the addition of chitosan gradually decreases the thermal stability of the materials, reaching approximately 275 °C for ELO/4CHI//OA, compared to the neat resin, which has *T*_5%_ = 297 °C. We conclude that the developed thermoset materials possess a very good thermal stability, and the addition of chitosan does not substantially influence this behaviour.

### 3.4. DMA Studies

The influence of the chitosan addition on the viscoelastic behaviour of the systems was studied using DMA. Table 5 shows the relevant thermomechanical parameters like storage modulus (*E*′), damping factor (tan *δ*), crosslink density (*ν*), and the mass between crosslinks (*M*_c_), which was calculated according to the Tobolsky [33] theory, as shown in the next equation:

$$Mc=\frac{3\rho RT}{E^{\prime }}$$

where *ρ* is the calculated density, *E*′ is the storage modulus in the rubbery region, *R* is the gas constant, and *T* is the absolute temperature.

**Figure 6:**
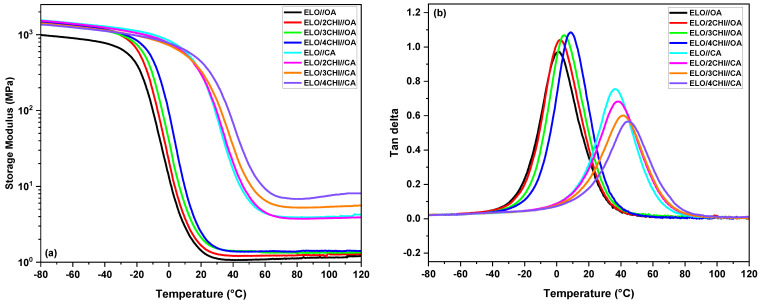
(**a**) Storage modulus and (**b**) damping factor vs. temperature for the biobased thermoset materials.

The graphical representation of the storage modulus (*E*′) vs. the temperature of the developed systems is displayed in Figure 6a, while the damping factor (tan *δ*) curves of the ELO/CHI//OA and ELO/CHI//CA systems are presented in Figure 6b.

First, it can be observed that the system crosslinked with CA is more rigid than the one with OA, having a storage modulus in the glassy region of ~1.4 GPa compared to 0.85 GPa. For the CA-based system, the *E*′ in the glassy region decreases slightly with the addition of chitosan, reaching around 1.2 GPa for the ELO/4CHI//CA system, while in the rubbery plateau, chitosan improves the mechanical properties by increasing the storage modulus of the final materials. In the case of the OA-based formulation, the chitosan addition improved the storage modulus in all the three regions, the *E*′ in the glassy plateau reaching about 1.3 GPa for the ELO/4CHI//OA material. The maximum value of the tan *δ* peak (*T*_α_) is associated with the glass transition temperature (*T*_g_) of the materials.

A similar trend of the thermomechanical properties of the materials is visible in the damping factor curves, plotted in Figure 6b.

Considering the crosslinking agent nature, the CA system presented higher tan *δ* values (*T*_α_ = 36 °C) compared to the ELO//OA materials (*T*_α_ = 0.7 °C). In both cases, the addition of chitosan strengthens the structure of the materials and increases the glass transition values of the systems; for the CA-based materials, the *T*_α_ transition is ranged in the interval from 36 °C (raw system) to 45 °C for the materials with 4CHI, while in the case of the OA-based systems, the *T*_α_ values are between 0.7 °C (neat resin) and 8.5 °C for the ELO/4CHI//OA system.

Regarding the intensity of the peak, in the case of CA systems, the height of the tan *δ* decreases with the increase in the amount of chitosan, confirming the increase in the stiffness of the materials. In the case of the OA systems, it can be observed that, although the glass transition increases with the increase in the amount of chitosan, the intensity of the tan *δ* peak increases. This fact indicates the double role of chitosan, namely strengthening the structure of the material as well as giving plasticity to the system to reduce the brittleness of the epoxy materials. The glass transition values obtained in this study are comparable with industrial ones such as Resinlab^®^ EP1200LV: *T*_g_ = 1 °C, Hexcel^®^ HexPly^®^ M27: *T*_g_ = 5 °C, or Epoxy Technology EPO-TEK^®^ E2036: *T*_g_ = 30 °C, which are used in a wide range of applications, such as semiconductors, electronics, or medical devices. The thermodynamic properties of the materials, as well as the beneficial effect of chitosan on them, was settled and confirmed by the values obtained for the crosslinking density (CA-based systems: *ν* = 0.4–0.84 mmol∙cm^−3^; OA-based systems: *ν* = 0.13–0.16 mmol∙cm^−3^) and the mass between crosslinks (CA-based systems: *M*_c_ = 1294–2622 g∙mol^−1^; OA-based systems: *M*_c_ = 7503–8085 g∙mol^−1^).

After an investigation of the specialized literature, we found that Xu et al. [34] obtained lower glass transitions for epoxy natural rubber and carboxymethyl chitosan (CMC)-based materials, which increased from −21.2 °C for pure epoxy natural rubber to −18.9 °C for that with 30 wt.% carboxymethyl chitosan. Moreover, the addition of CMC led to a substantial increase in the storage modulus in the rubbery plateau from 1.4 MPa to 10.2 MPa.

The stiffness of the systems was evaluated through Shore hardness testing and the obtained values (Table 5) show similar behaviour as in the DMA results. The results obtained for the oxalic acid systems reveal a medium hardness of the materials (“medium hard” category), obtaining a value of about 60 SA, while the citric acid system is harder, being included in the “hard materials” category with a hardness of 91 SA. It can be seen that both systems showed a higher penetration resistance with the addition of chitosan, reaching up to 67 SA in the case of ELO/4CHI//OA and up to 98 SA in the case of ELO/4CHI//CA, the obtained results being similar with the commercial petro-based resins (Abatron AboCast 8110-4/AboCure 8110-3 = 15–94 SA; Kohesi Bond KB 1048 FL = 60–70 SA; Master Bond EP51FL-1ND-2 = 50–60 SA)

Therefore, the loading of bio-based epoxy resins with chitosan improves the mechanical properties of the materials while simultaneously giving them plasticity, which reduces the brittle nature of epoxy systems.

### 3.5. Tensile Testing

**Figure 7:**
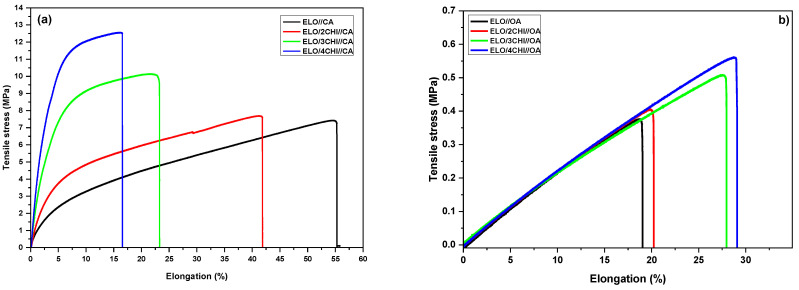
Tensile properties of the (**a**) ELO/CHI//CA and (**b**) ELO/CHI//OA systems.

The mechanical properties of the developed materials were investigated through tensile testing, the graphical representation of the tensile stress relative to elongation being displayed in Figure 7. Parameters like Young’s modulus, tensile strength and stress, and energy at break were determined and tabulated in Table 6.

As can be seen, the system crosslinked with CA showed a higher elongation at break (*ε*~55%) compared to the OA-based formulation (*ε*~19%), being approximately three times higher. In the case of systems with CA, the addition of chitosan led to a gradual reduction of the elongation at break of the materials, reaching approximately 17% for the system with 4CHI; for the OA formulations, the addition of chitosan led to a slight increase in the strain at break from 19% for the raw material to ~29% for ELO/4CHI//OA.

CA-based systems also showed higher values for tensile strength and Young’s modulus than OA formulations. These results correlate very well with the DMA data, where CA systems, also, had much higher crosslink densities. Based on the obtained data, the tensile stress of the ELO//CA materials is about 20 times higher than that of ELO//OA formulation. In both cases, the chitosan addition improved the mechanical properties, the tensile stress of the CA-based systems ranging from 7.4 to 12.5 MPa, and for OA-based materials between 0.38–0.53 MPa. Regarding Young’s modulus, the values obtained depending on the hardener nature were totally different; the system crosslinked with CA had a modulus of approximately 94 MPa, while the Young’s modulus of the material developed with OA decreased by approximately 40 times (2.39 MPa). The addition of chitosan improved the mechanical properties of both formulations, but the increase in the Young’s modulus of the systems with CA increased substantially (from 93 MPa for raw resin to 404 MPa for ELO/4CHI//CA), compared to the one with OA where the increase was smaller (from 2.39 to 2.75 MPa). Parameters such as the specific modulus, specific strength and specific length were determined and analysed to better integrate the designed materials in specific industrial areas. The strength of the systems was also determined by calculating the brittleness (*B*) of the materials using the equation determined by Brostow et al. [35,36,37]:

$$B=1/\varepsilon_{b}E^{\prime }$$

where *ε_b_* is the elongation at break and *E*′ represents the storage modulus calculated using DMA at a frequency of 1.0 Hz. It is known that the lower the *B* value, the lower the brittleness of the material, so that materials with OA are much more fragile than those with CA; also, in the case of materials with CA, the addition of chitosan slightly increases the brittleness, while in the case of systems with OA, chitosan decreases the fragility and improves the materials. The two types of systems show significant differences in tensile strength and Young’s modulus values due to the nature of carboxylic acid and the CHI contribution. From Figure 7, we can observe that the addition of chitosan has an opposite effect on elongation at break if we compare both systems. This result is in strong correlation with the networks crosslinking densities (Table 5) that are, for example, five times higher in the thermosets based on CA, which also have the M_c_~5.5 times lower. This effect is similarly correlated with the Young’s modulus values that are low and almost constant for the OA based systems (~2.5 MPa) but increase with CHI content, up to 161 time higher in the materials based on CA (more rigid networks).

**Table 6:** Mechanical parameters of ELO-based materials [38].

Sample	Young’s Modulus (MPa)	Tensile Stress (MPa)	Elongation at Break (%)	Specific Modulus *E*/ρ (10^6^·m^2^/s^2^)	Specific Strength σ/ρ (kN·m/kg)	Specific Length σ/ρ·g (km)	*B* (%·Pa/10^10^)	Energy at Break(J)
ELO//OA	2.39 ± 0.13	0.38 ± 0.03	18.6 ± 0.05	0.0023	0.37	0.037	44.42	0.02
ELO/2CHI//OA	2.43 ± 0.06	0.4 ± 0.02	20 ± 0.03	0.0022	0.36	0.037	35.96	0.02
ELO/3CHI//OA	2.51 ± 0.07	0.48 ± 0.03	27.7 ± 0.04	0.0021	0.41	0.042	21.37	0.02
ELO/4CHI//OA	2.75 ± 0.12	0.53 ± 0.04	28.8 ± 0.06	0.0023	0.45	0.046	19.06	0.03
ELO//CA	93.3 ± 28.81	7.4 ± 0.02	54.9 ± 0.5	0.09	7.05	0.72	0.11	0.96
ELO/2CHI//CA	137.10 ± 35.36	7.8 ± 0.06	41.6 ± 0.7	0.13	7.4	0.76	0.14	1.09
ELO/3CHI//CA	285.04 ± 28.22	10.1 ± 0.7	22.4 ± 0.3	0.27	9.4	0.96	0.20	0.87
ELO/4CHI//CA	404.35 ± 15.84	12.5 ± 0.04	16.4 ± 0.4	0.38	11.6	1.18	0.19	0.61

The reported studies show that Jabeen et al. [24] obtained thermosetting materials based on chitosan. For the neat chitosan film, the tensile strength was about 61 MPa, with an elongation at break of 16%. When they added bisphenol F of diglycidyl ether, they observed an increase in the elongation to 31% and a decrease in tensile strength to 45 MPa. The enhancement of the elongation was due to the covalent bonding between the amino groups of the chitosan structure and the epoxy groups, producing an intrachain network. In another study, Xu et al. [34] reported the development of elastomers based on natural rubber epoxy (ENR) and carboxymethyl chitosan (CMC). The tensile stress values of the systems increased from 170% (non-ENR) to 370% for the material with 20 wt.% CMC, but then decreased to 210%. The materials showed the same trend for tensile stress: it increased from 0.5 MPa to 2.6 MPa and then decreased to 2.3 MPa. Also, Ding et al. [32] show the effect of different carboxylic acids on the mechanical properties of the thermosetting resins obtained from ELO. The mechanical properties were similar in terms of tensile strain, with higher values between 1.1 MPa and 3.8 MPa for tensile stress, and with values between 4.7 MPa and 10 MPa for Young’s modulus. Their adipic acid sample showed the best mechanical properties: tensile strength of 8.8 MPa, elongation at break of 55% and Young’s modulus of 22 MPa. Also, the biobased systems developed in this work present similar properties to the commercial ones such as Master Bond EP110F8-5 (σ = 6.89–13.8 MPa; ε = 40–60%), Resinlab^®^ EP1200LV (σ = 6.89 MPa; ε = 27%), and Henkel Loctite^®^ Stycast^®^ ES 0626 (σ = 7.52 MPa; ε = 40–50%), thus being a good alternative for materials based on petroleum derivatives.

Therefore, fusing into a single molecule a rigid backbone such as chitosan, with flexible side chains such as ELO, generates materials with improved mechanical properties.

## 4. Conclusions

In this study, new fully biobased thermoset networks were developed starting from an epoxidized vegetable oil, such as ELO, which was crosslinked with two different acids: oxalic and citric acid. The influence of the addition of chitosan into the thermoset structure was studied. The thermomechanical properties of the designed biomaterials, studied using DMA, increased in relation to the chitosan content. The storage modulus in the case of the CA-based systems decreased slightly in the glassy region (from 1.4 GPa to 1.2 GPa), but in the rubbery region, the *E*′ value doubled (from 4 MPa to 8 MPa). Regarding the systems with OA, the storage modulus increased both in the glassy (from 0.8 GPa to 1.3 GPa) and rubbery (from 1.2 MPa to 1.4 MPa) regions in relation to the amount of biopolymer. The beneficial effect on the mechanical properties of the materials arising from the addition of chitosan was also confirmed by the tensile tests. A considerable improvement can be seen in the case of systems with CA, where the tensile stress started from 7.4 MPa for the neat resin and reached 12.5 MPa for the system loaded with 4 wt.% chitosan, while the Young’s modulus increased from 94 MPa to 404 MPa.

In conclusion, by combining a vegetable oils-based epoxy monomer with waste chitosan from the seafood industry and natural oxalic or citric acids, 100% biobased epoxy thermoset materials were designed. By varying the nature of the hardener, as well as the amount of biopolymer, the properties of such systems can be modulated and adapted to different fields of application as parts of medical devices, matrices for composites for automotive components, flooring, and coatings for wood protection.

## Figures and Tables

**Table 1:** Sample composition and labelling.

Ratio ELO/acid	wt.% CHI	Acronyms
R = 1/1	0	ELO//O(C)A
R = 1/1	2	ELO/2CHI//O(C)A
R = 1/1	3	ELO/3CHI//O(C)A
R = 1/1	4	ELO/4CHI//O(C)A

**Table 2:** FT-IR assignments characteristic bands of crosslinked mixtures.

Group	Absorption Peak (cm^−1^)
–OH	3560
–CO vibration of the ester linkage	1740
–CO vibration of the ester and ether linkages formed during curing)	1168, 1069
-Methylene (–CH_2_–)	2926, 2851

**Table 3:** DSC data obtained during crosslinking evaluations.

Systems	Δ*H* (J·g^−1^)	*T*_peak_ (°C)	*T*_onset_–T_end_ (°C)
ELO//OA	50	87	56–130
ELO/2CHI//OA	83	85	37–130
ELO/3CHI//OA	84	84	32–138
ELO/4CHI//OA	107	82	34–130
ELO//CA	125	131	98–178
ELO/2CHI//CA	138	128	100–179
ELO/3CHI//CA	152	129	100–182
ELO/4CHI//CA	173	127	94–192

**Table 4:** TGA parameters for the ELO-based systems loaded with chitosan.

Systems	*T*_5%_ (°C)	*T*_30%_ (°C)	*T*_dmax_ (°C)
ELO//OA	297	380	399
ELO/2CHI//OA	286	371	391
ELO/3CHI//OA	282	369	397
ELO/4CHI//OA	275	368	391
ELO//CA	283	375	392
ELO/2CHI//CA	283	375	392
ELO/3CHI//CA	282	375	392
ELO/4CHI//CA	276	367	391

**Table 5:** Thermomechanical properties of ELO/CHI//OA and ELO/CHI//CA thermosets.

Sample	Density (g/cm^3^)	Shore Hardness SA	*E*′ Glassy Region (−50 °C) (MPa)	*E*′ Rubbery Region (100 °C) (MPa)	T_α_ (°C)	tan *δ* Peak Amplitude	*ν* (mmol∙cm^−3^)	*M*_c_ (g/mol)
ELO//OA	1.04	60	852	1.2	0.7	0.97	0.13	8085
ELO/2CHI//OA	1.11	63	1210	1.3	2	1.04	0.14	7792
ELO/3CHI//OA	1.17	65	1240	1.35	4.6	1.07	0.15	7563
ELO/4CHI//OA	1.18	67	1260	1.4	8.5	1.09	0.16	7503
ELO//CA	1.05	91	1351	4	36	0.76	0.40	2622
ELO/2CHI//CA	1.06	92	1325	3.8	38	0.68	0.42	2521
ELO/3CHI//CA	1.07	95	1217	5.4	42	0.60	0.57	1870
ELO/4CHI//CA	1.08	98	1186	7.6	45	0.57	0.84	1294

## Data Availability

Data are contained within the article and Supplementary Materials.

## Supplementary Materials

The following supporting information can be downloaded at: https://www.mdpi.com/article/10.3390/polym15244627/s1, Figure S1. Alkaline deacetylation of chitin; Figure S2. Chemical structures of the reactants; Figure S3: FT-IR spectra of (a) chitosan, (b) ELO, (c) OA (oxalic acid), and (d) CA (citric acid); Table S1. FT-IR assignments for the characteristic bands of ELO, CHI, OA and CA compounds; Figure S4: FT-IR spectra for crosslinked bio-resins; Figure S5: Dynamic DSC thermograms for ELO//OA systems with different percentage of chitosan; Figure S6: TGA and DTG curves of the raw chitosan; Figure S7. Physical appearance of the biobased thermosets.
