# Supplementary material for: Valorisation of Chitosan Natural Building Block as a Primary Strategy for the Development of Sustainable Fully Bio-Based Epoxy Resins
Source: Polymers (Basel). 2023 Dec 6;15(24):4627. doi: 10.3390/polym15244627 (PMC10747223; doi:10.3390/polym15244627)
Supplement: Supplementary file 1 [file polymers-15-04627-s001.zip › polymers-2735621-supplementary.pdf]

Supporting Information

Valorisation of Chitosan Natural Building Block as a Primary Strategy for the Development of Sustainable Fully Bio-Based Epoxy Resins

Iolanda Fusteș-Dămoc [1,2], Roxana Dinu [1], Teodor Măluțan [2,*] and Alice Mija [1,*]

[1] University Côte d’Azur, Institute of Chemistry of Nice (ICN), UMR CNRS 7272, 06108 Nice, France
[2] “Cristofor Simionescu” Faculty of Chemical Engineering and Environmental Protection, “Gheorghe Asachi” Technical University of Iasi, 73 Prof. D. Mangeron Street, 700050 Iasi, Romania

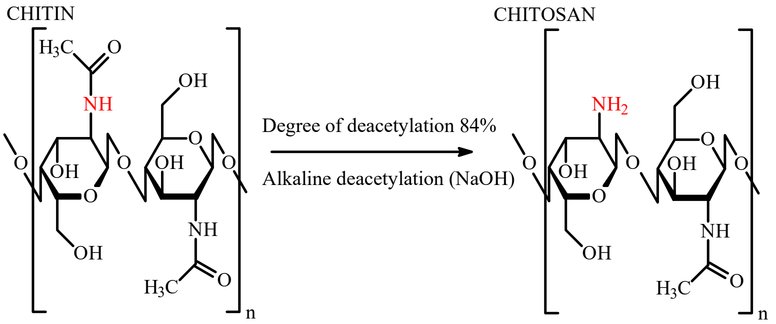

Figure S1. Alkaline deacetylation of chitin.

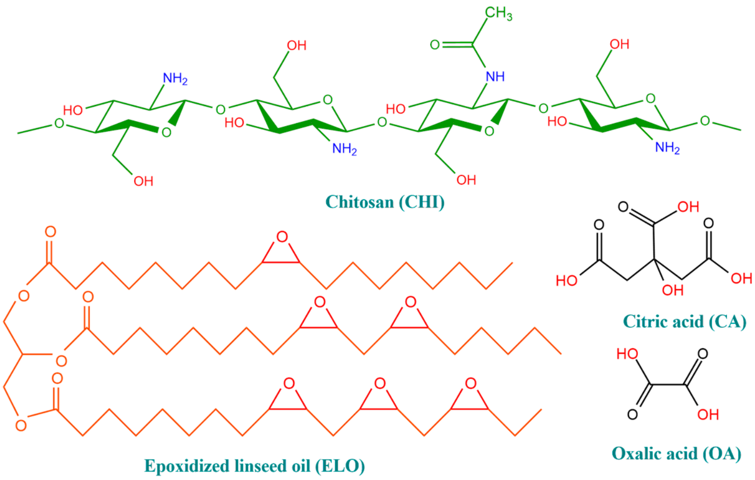

Figure S2. Chemical structures of the reactants.

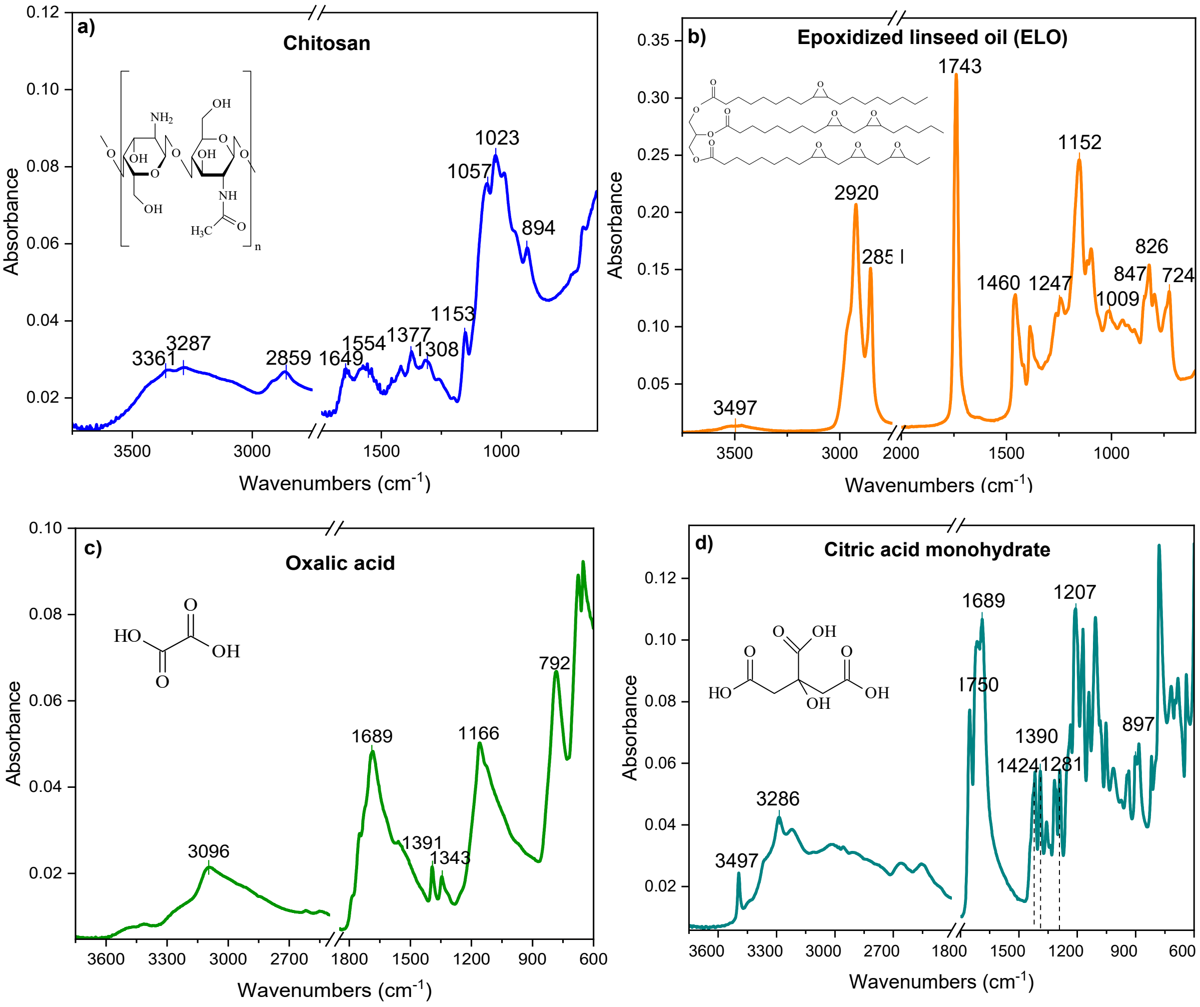

Figure S3. FT-IR spectra of a) chitosan, b) ELO, c) OA (oxalic acid), and d) CA (citric acid).

Table S1. FT-IR assignments for the characteristic bands of ELO, CHI, OA and CA compounds.

**Monomers**	**Group**	**Absorption peak (cm^{-1})**
ELO	–O–H stretching (weak)	3497
	–C–H, asymmetric stretching of CH_2, CH_3	2920
	–C–H symmetric stretching of CH_2, CH_3	2851
	Triglyceride carbonyl of ester C=O	1743
	Scissoring of CH_2, asymmetric bending of CH_3	1508
	$-CH_3$ symmetric deformational	1383
	C–O–C stretching of epoxy groups	1247
	C–O asymmetric of ester groups (O=C–O)	1152
	C–O stretching of O–CH_2	1098

	Oxirane ring, C–O or C–H wagging of epoxy groups	847-826
Citric acid	O–H stretching (COOH), H bonding	3500-2500
	C=O stretching mode in the carboxylic dimer	1750, 1689
	scissoring type $–CH_2$ bending vibration	1424
	C–O–C scissoring vibration	1390
	C–O vibrations	1281, 1207,1105
	COOH	897
Oxalic acid	–OH stretch, H bonding	3430-2500
	C=O stretching	1689
	β (OH) symmetric stretching	1343
	–OH asymmetric stretching	1166
	COOH	792
Chitosan	N–H, O–H stretching, H bonding	3361, 3287
	C-H stretching	2859
	Amide band I C=O	1649
	Amide band II $–NH_2$ due to deacetilation	1554
	Amide band III- C–N	1377
	bridge –O– stretch; Saccharide structure of chitosan C–O–C	1153
	C–O stretch, secondary hydroxyl group	1057
	C–O stretch, primary hydroxyl group	1023
	Pyranose ring	894

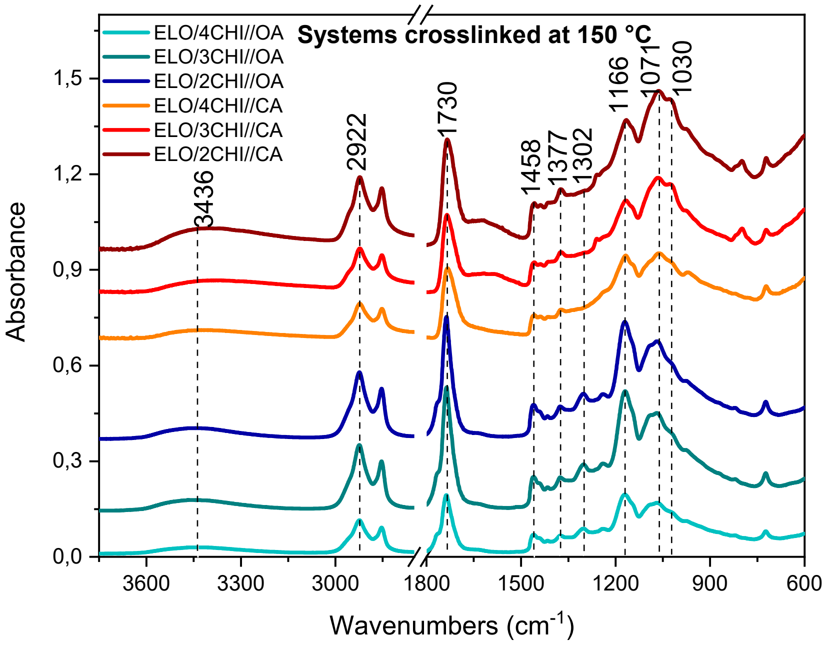

Figure S4. FT-IR spectra for crosslinked bio-resins.

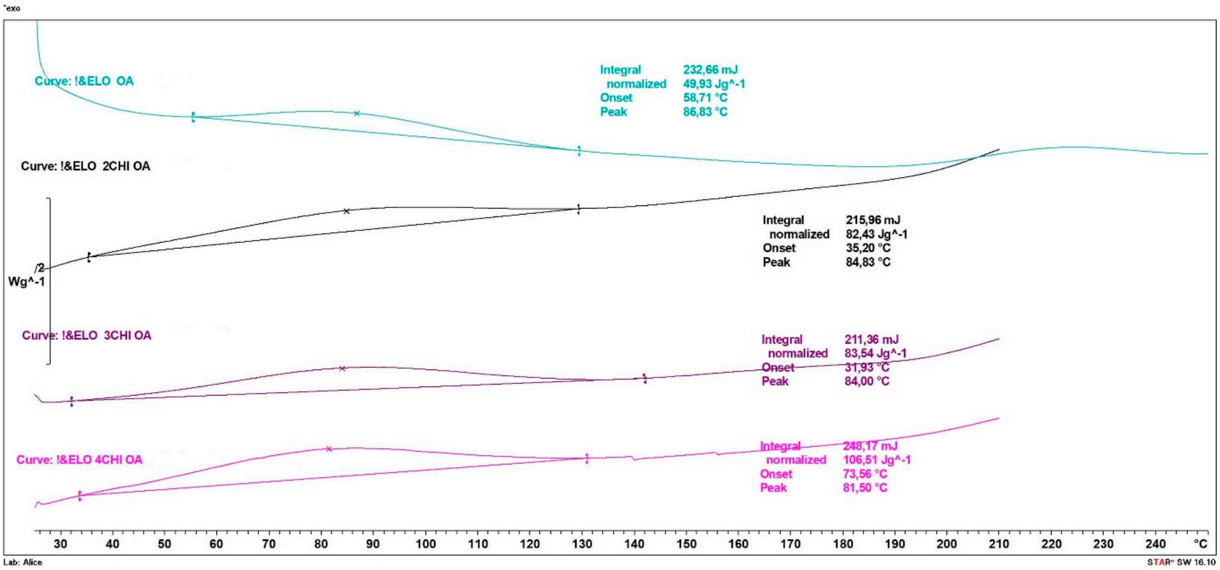

Figure S5. Dynamic DSC thermograms for ELO//OA systems with different percentage of chitosan

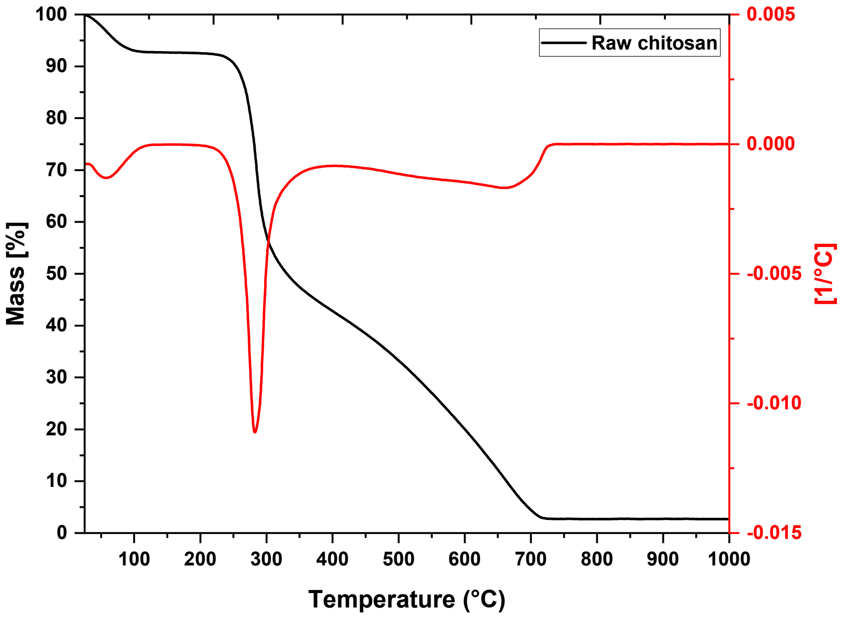

Figure S6. TGA and DTG curves of the raw chitosan.

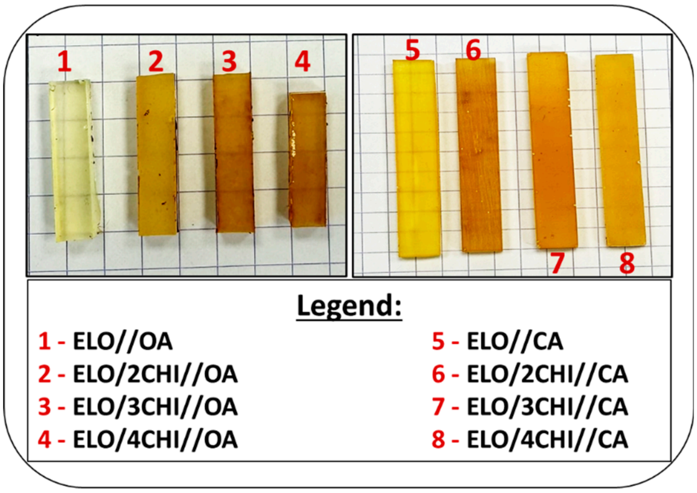

Figure S7. Physical appearance of the biobased thermosets
